# The Effect of Coasting on Intracytoplasmic Sperm Injection Outcome in Antagonist and Agonist Cycle 

**DOI:** 10.22074/ijfs.2016.5144

**Published:** 2016-11-11

**Authors:** Z.Candan İltemir Duvan, Müberra Namlı Kalem, Yuksel Onaran, Esra Aktepe Keskin, Aylin Ayrım, Aslıhan Pekel, Hasan Kafalı, Nilgün Turhan

**Affiliations:** 1 Department of Obstetrics and Gynaecology, School of Medicine, Turgut Ozal University, Ankara, Turkey; 2 Department of IVF-Embriology, School of Medicine, Turgut Ozal University, Ankara, Turkey; 3 Department of Obstetrics and Gynaecology, School of Medicine, Gazi University, Ankara, Turkey; 4 Department of Obstetrics and Gynaecology, School of Medicine, Muğla Sıtkı Koçman University, Muğla, Turkey

**Keywords:** Ovarian Hyperstimulation Syndrome, GnRH Agonist, GnRH Antagonist

## Abstract

**Background:**

Coasting can reduce the ovarian hyperstimulation syndrome (OHSS) risk in ovulation induction cycles before intracytoplasmic sperm injection (ICSI). This study aimed to investigate the effect of gonadotropin-releasing hormone (GnRH) agonist and GnRH antagonist protocols to controlled ovarian hyperstimulation (COH) cycles with coasting on the parameters of ICSI cycles and the outcome.

**Materials and Methods:**

In a retrospective cohort study, 117 ICSI cycles were per- formed and coasting was applied due to hyperresponse, between 2006 and 2011. The ICSI outcomes after coasting were then compared between the GnRH agonist group (n=91) and the GnRH antagonist group (n=26).

**Results:**

The duration of induction and the total consumption of gonadotropins were found to be similar. Estradiol (E_2_) levels on human chorionic gonadotropin (hCG) day were found higher in the agonist group. Coasting days were similar when the two groups were compared. The number of mature oocytes and the fertilization rates were similar in both groups; however, the number of grade 1 (G1) embryos and the number of transferred embryos were higher in the agonist group. Implantation rates were significantly higher in the antagonist group compared to the agonist group. Pregnancy rates/embryo transfer rates were higher in the antagonist group; however, this difference was not statistically significant (32.8% for agonist group vs. 39.1% for antagonist group, P>0.05).

**Conclusion:**

The present study showed that applying GnRH-agonist and GnRH-antago- nist protocols to coasted cycles did not result in any differences in cycle parameters and clinical pregnancy rates.

## Introduction

Ovarian hyperstimulation syndrome (OHSS) is the most important and potentially life-threatening iatrogenic complication of ovulation induction (1). Coasting is the stopping of gonadotropin administration when OHSS risk develops during controlled ovarian hyperstimulation (COH) and the withholding of human chorionic gonadotropin (hCG) administration until Estradiol (E_2_) levels reach a plateau or drop to a safe range with a significant reduction (2). Follicular growth is generally correlated with follicle-stimulating hormone (FSH) threshold. Large follicles are more resistant to apoptosis and atresia; thereby, larger follicles continue developing while immature follicles undergo a selective regression when the FSH level drops. Coasting works with this principle to reduce the functional granulosa cell mass available for luteinization and prevent an increase in vasoactive substances involved in OHSS pathogenesis (3). Coasting does not completely eliminate the OHSS risk in high-risk patients, but may reduce incidence and OHSS severity (4, 5). 

OHSS risk is lower in gonadotropin-releasing hormone (GnRH) antagonist cycles, so this protocol should be preferred in high-risk patients. Shifting from agonist protocols to antagonist protocols is a good alternative for preventing OHSS since it ensures the proper maintenance of granulosa functions (6). Coasting in agonist cycles has been in use since the 1980s (7), while there is a broad range of publications about its outcomes (8). Coasting in antagonist cycles was started in 2001 as case reports (9), and coasting was shown to have no adverse effects on *in vitro* fertilization (IVF) outcome nor in antagonist cycles in subsequent studies (10).

In a study by Farhi et al. (11), they compared coasting practices in agonist and antagonist cycles and did not find any difference in cycle parameters, number of retrieved oocytes or pregnancy rates. They have also reported that the same coasting criteria could be applied to agonist and antagonist protocols. In a study by Tarlatzis et al. (12), they have reported a lower pregnancy rate in agonist cycles with coasting as compared to agonist cycles.

The present study aimed to investigate the effect of applying agonist or antagonist protocols to COH cycles with coasting on the parameters of intracytoplasmic sperm injection (ICSI) cycles and the outcome.

## Materials and Methods

The present retrospective cohort study was conducted by the IVF Unit of Turgut Ozal University, Ankara, Turkey. During the study period, 1140 ICSI cycles were performed in our IVF center. Coasting was applied for 117 (11%) cycles, meaning in 92 (78.6%) cycles after GnRH-agonist protocol and in 26 (22.2%) cycles after GnRH-antagonist protocol. The study investigated retrospectively 117 cycles (11% of total) in this unit between 2006 and 2011, in which ICSI was performed and coasting was applied due to hyperresponse. Cycles were divided into two following groups according to the preferred stimulation protocol: i. GnRH agonist group (n=91) and ii. GnRH antagonist group (n=26). GnRH agonist protocol was initiated with leuprolide acetate 1 mg daily (Lucrin, Abbott, Turkey) in midluteal phase. Down-regulation was confirmed after 13-15 days (no ovarian cysts>18 mm, E_2_ <50 pg/mL) that was followed by gonadotropin stimulation. After down regulation, the dose of leuprolide acetate was reduced to 0.5 mg daily until hCG day. The antagonist protocol consisted of daily gonadotropin stimulation from day 3 or 4 of menstruation followed by daily injections of Cetrotide 0.25 mg (Serono, Switzerland) or Orgalutran 0.25 mg (N.V. Organon, The Netherlands) once the leading follicle reached 14 mm and until the day of hCG injection. 

Gonadotropin stimulations was performed by recombinant FSH (rFSH), as follitropin-alfa (Gonal F, Merck- Serono, Switzerland) or follitropin-beta (Puregon, N.V. Organon, Oss, The Netherlands), or in combination with urinary gonadotropins (Menogon, Ferring, Germany). The choice of agonist or antagonist protocol for COH was made according to the patient characteristics [age, antral follicle count (AFC), and body mass index (BMI)] or previous IVF cycles responses if available. The following infertility factors were found in patients undergoing coasting: 45% male factors, 24% polycystic ovary syndrome (PCOS), 16% unexplained infertility, and 15% others.

The study included women aged <38 years who underwent ovarian stimulation for ICSI with the GnRH agonist down-regulation protocol or GnRH-antagonist protocol and were subsequently coasted for risk of severe OHSS. Azoospermia and premature ovarian insufficiency were the exclusion criteria. Being retrospective in nature with anonymous data collection, this study did not require Ethical Committee approval. A written consent form was signed by all participants.

Throughout the treatment, the ovarian response was evaluated by measuring follicles via transvaginal ultrasound and measuring serum levels of E_2_ once every 1 to 3 days from day 4 of stimulation. The treatment was maintained by adjusting the gonadotropin dose according to these outcomes.

Coasting was applied to COH cycles when the serum E_2_ concentration was 4,000 pg/mL or when at least 20 follicles, each measuring 10 mm in diameter and 20% measuring 15 mm in diameter, were present (7). The minimum coasting days was 1.7 ± 1.1, and the maximum coasting days was 4.1 ± 1.0.

Cycles were canceled if coasting period was more than 4 days. During the coasting period, GnRH antagonist in antagonist protocol and leuprolide acetate in agonist protocol were administered in the same dosage until the E_2_ concentrations dropped. When the E_2_ concentration dropped below 4,000 pg/ mL or when at least two follicles reached 18 mm in diameter, ovulation was triggered by administration of 5,000/10,000 IU hCG (Pregnyl, Organon, The Netherlands). Since E_2_ levels were not decreased during the coasted period, 7.6 % of cycles (9 cycles) were abandoned in the study.

Oocyte retrieval was performed 35-36 hours after hCG trigger. Oocytes were fertilized by ICSI. After oocyte retrieval, all patients were prophylactically administered 50 mL human albumin intravenous (Human Albumin, Octapharma, Germany). Three days after oocyte retrieval, embryos were transferred transcervically under ultrasound control. Luteal phases were supported by micronized progesterone 200 mg three times a day (Progestan, Koçak, Turkey). Clinical pregnancy was defined by a demonstrable gestational sac accompanied by fetal heart activity on ultrasound.

### Statistical analysis

The data were analyzed using the Statistical Package for the Social Sciences (SPSS, SPSS Inc., USA) for Windows 11.5 software package. The compatibility of discrete and continuous numeric variables with normal distribution was analyzed using the Shapiro-Wilk test. Descriptive statistics were expressed in mean ± SD or median (minimum-maximum) for discrete and continuous variables, while categorical variables were expressed in number of cases (N) and percentage (%). The significance of the difference between the groups was analyzed using Student’s t test, and the significance of the difference for mean values was analyzed using the Mann-Whitney U-test. Categorical variables were evaluated using Pearson’s Chi-squared or Likelihood ratio Test. The value of P<0.05 was considered statistically significant.

## Results

Agonist protocol was applied to 91 patients and antagonist protocol was applied to 26 patients in the present study. There were no significant differences noted for BMI, duration of infertility, basal FSH and E_2_ levels in groups. In the antagonist group, patients’ ages were significantly higher compared to the agonist group. Ovarian volume was significantly higher in the agonist group compared to the antagonist group (Table 1). There is a significant difference in the age of women in both groups, as this could affect selection in treatment method, which is applied in our daily practice. The significant difference in ovarian volume is declared by the age difference and the high rate of PCOS patients in the agonist group.

**Table 1:** Demographic characteristics and basal hormonal parameters of both groups


Characteristic	Agonist group	Antagonist group	P value

Age (Y)	29.3 ± 4.6	31.6 ± 4.3	0.024*
BMI (kg/m^2^)	24.2 ± 4.5	23.1 ± 3.7	0.277
Duration of infertility (Y)	5.3 ± 3.7	4.5 ± 3.9	0.311
FSH (mIU/mL)	6.8 ± 1.2	8.1 ± 1.1	0.540
Basal E_2_ level (pg/mL)	38 ± 22.9	48.1 ± 19.7	0.057
Ovarian volume (cm^3^)	16.6 ± 10.6	9.8 ± 7.9	0.001*
			


*; Significant at P<0.005, FSH; Follicle-stimulating hormone, E_2_; Estradiol, and BMI; Body mass index.

Cycle characteristics and cycle outcomes of both groups are presented in Table 2. Initiation dose of gonadotropin (220.9 ± 64.1 vs. 193.7 ± 72.3), duration of induction (8.4 ± 3.8 vs. 8.9 ± 2.9), and total consumption of gonadotropin (1793 ± 830 vs. 2004.3 ± 1677.6) were similar between the groups. The mean serum E_2_ levels on day of hCG were significantly higher in the agonist group compared to the antagonist group (3950.2 ± 300.3 vs. 3600 ± 250.2, P<0.05). Endometrial width on day of hCG was also similar between the groups (10 ± 2.1 and 9.7 ± 2.0).

Coasting days were found to be similar for both groups (3.1 ± 1.0 and 2.8 ± 1.1). There was no sig- nificant difference in the number of mature (M2) oo- cytes and the number of 2-pronucleus (PN) embryos (8.7 ± 3.8 vs. 9.8 ± 3.6 and 6.7 ± 3.2 vs. 7.8 ± 3.1, respectively). However, the number of grade 1 (G1) embryos and the number of transferred embryos were significantly lower in the antagonist group compared to the agonist group (4 ± 2.2 vs. 3.8 ± 2, P<0.05 and 2.4 ± 0.8 vs. 1.6 ± 0.7, P<0.05, respectively).

There was no significant difference in fertilization rates between two groups (72.5 ± 22.4 vs. 79.6 ± 19.7). However, implantation rates were significantly higher in the antagonist group compared to the agonist group (19.7 vs. 26.3, P<0.05). Pregnancy rates per embryo transfer were found to be similar in both groups (32.8 vs. 39.1).

**Table 2:** Cycle characteristics and outcome for coasted cycles


	Agonist group	Antagonist group	P value

Initial dose of rFSH (IU)	220.9 ± 64.1	193.7 ± 72.3	0.060
Duration of induction (day)	8.4 ± 3.8	8.9 ± 2.9	0.773
E level on hCG day (pg/ml)2	3950.2 ± 300.3	3600 ± 250.2	0.044*
Endometrial width (mm)	10 ± 2.1	9.7 ± 2.0	0.662
Total consumption of gonadotrophins	1793 ± 830	2004.3 ± 1677.6	0.571
Coasting days	3.1 ± 1.0	2.8 ± 1.1	0.736
M2 oocytes (n)	8.7 ± 3.8	9.8 ± 3.6	0.180
Transfer day	3 ± 1.1	3.0 ± 1.0	0.530
G1 embryo (n)	4 ± 2.2	3.8 ± 2	0.024*
Number of transferred embryos (n)	2.4 ± 0.8	1.6 ± 0.7	0.001*
Fertilization rate (%)	72.5 ± 22.4	79.6 ± 19.7	0.134
Implantation rate (%)	19.7	26.3	0.008*
Pregnancy rate/embryo transfer (%)	32.8	39.1	0.057


*; Significant at P<0.005, G1; Number of grade 1 embryo, hCG; Human chorionic gonadotropin, E_2_ ; Estradiol, rFSH; Recombinant follicle-stimulating hormone, and M2; Number of mature oocytes.

## Discussion

The present study, which investigated whether applying coasting to agonist and antagonist cycles had any differences, found no significant differences in cycle characteristics and clinical pregnancy rates. The M2 oocytes and the fertilization rates were similar in both groups; however, the number of G1 embryos and the number of transferred embryos were higher in the agonist group. Implantation rates were significantly higher in the antagonist group compared to the agonist group. Pregnancy rates/embryo transfer rates were higher in the antagonist group; however, this difference was not statistically significant. 

The higher age in the antagonist protocol group is an indicator of a tendency toward antagonist protocol with increasing age at the clinical decision stage. This opinion was shared by the articles of Lainas et al. (13) and Al-Inany and Aboulghar (14). The lower ovarian volume of the antagonist group compared to the agonist group is a result of the fact that ovarian volume decreases with increasing age (15). 

The present study found similar initial rFSH doses and total consumptions of gonadotropin in both groups, which is consistent with the study by Huirne et al. (16). There was no significant difference in induction times for both protocols. However, a number of studies have reported that induction times are generally shorter in antagonist protocol (14, 17). Our hyperresponsive patients may experience shorter induction time in agonist protocol.

The present study found higher E_2_ levels on hCG day in the agonist group. The studies by Farhi et al. (11) and Tarlatzis et al. (12) comparing agonist and antagonist coasted cycles reported lower E_2_ levels on hCG day in the antagonist groups. Similarly, Elter et al. (18) investigated the pattern of E_2_ change in agonist and antagonist coasting protocols and found lower E_2_ levels on hCG day in antagonist cycles.

There are several studies about the coasting time in cycles coasted due to OHSS risk; however, the optimal coasting time that would not affect the pregnancy rate has not yet been defined (19, 20). It is recommended that the coasting time to be no more than four days (21, 22). In the present study, coasting times did not exceed 4 days and no difference was found in coasting time between two groups. 

The number of post-coasting mature oocytes was found similar in the agonist and antagonist groups. The study by Elter et al. (18) obtained a higher number of mature oocytes in the antagonist coasting group compared to the agonist group. The authors attributed this finding to the shorter coasting time in the antagonist group. Farhi et al. (11) compared the number of collected oocytes and the coasting days together and showed that a coasting time of more than 3 days reduced the number of mature oocytes in the antagonist group. They demonstrated that the number of oocytes was reduced when the coasting time was 1-2 days in the agonist group, whereas it did not change when the time was 4 days and more.

The present study found that there was no difference in fertilization rates between agonist and antagonist coasting groups. In the studies by Bahceci et al. (10) and Mansour et al. (22), fertilization rates did not differ in antagonist coasting cycles compared to control groups. Likewise, in the studies by Farhi et al. (11) and Tarlatzis et al. (12), they compared agonist and antagonist coasting cycles and found no significant difference in fertilization rates. 

Although there were no differences in the number of mature oocytes and the fertilization rates between two groups in the present study, the number of G1 embryos and the number of transferred embryos were higher in the agonist group. A similar study in the literature reported that the quality of embryos was not affected by the protocol applied to the coasting cycles, but an extreme drop in the E_2_ level and the prolonged duration of this condition were shown to affect the quality of embryos (10, 11, 23).

The studies on pregnancy rate investigated coasting practices in agonist and antagonist protocols individually, while most of them showed no effect on implantation and pregnancy rates after coasting. Their finding reported that the implantation and pregnancy rates are reduced only in the cycles with a coasting time exceeding 4 days, regardless of the protocol used (10, 22, 24). The study by Grace et al. (25) compared the cycles with and without coasting and reported that coasting reduced the fertilization rates, the quality of embryos and pregnancy rates. The study by Farhi et al. (11) comparing agonist and antagonist coasting did not find any difference in pregnancy rates, and Tarlatzis et al. (12) found a lower pregnancy rate in the antagonist coasting group than the agonist coasting group. In the present study, the implantation rates were significantly higher in the antagonist group, whereas the pregnancy rates/embryo transfer rates were also higher in the antagonist group, indicating there were no statistically significant in this regard. The increased implantation rates in the antagonist group may be attributed to the impaired endometrial receptivity due to the higher levels of E_2_ in the agonist group.

The present study is one of the rare studies investigating the effect of a selected protocol on the cycle outcome in coasted cycles. The most important drawbacks of the study were the differences between the participants in the groups, leading to bias in the study. The reason of this difference mostly depended on our preferences, as we used GnRH agonist protocols in ICSI cycles routinely till 2009. After that time, we increased our experience with GnRH-antagonist protocol, and we then preferred one of these protocols, according to patients’ characteristics. Another important limitations of the present study were its retrospective nature and the lack of a control group without coasting. The present study may indicate the OHSS rates in the selected protocols, and at which rates coasting was required in agonist and antagonist cycles. The present study was also limited by not evaluating the effect of E_2_ drop rate on cycle outcome during the coasting (8) and the correlation between the coasting days and the number of oocytes obtained (26), which are considered as controversial topics in the literature. In addition, we wanted to declare that in all cases of infertility, whether of female, male or “unexplained” nature, regardless of sperm function, ICSI bypasses most dysfunctions, eliminating the majority of barriers to fertilization. As the compelling evidence of ICSI, we prefer ICSI procedure routinely in all cases rather than IVF.

## Conclusion

The present study showed that applying GnRH-agonist or GnRH-antagonist protocols to coasted cycles did not result in any differences in cycle parameters and clinical pregnancy rates. It is suggested that future prospective randomize con- trolled studies about coasted cycles in IVF.
